# Oral Changes in Hospitalized COVID-19 Patients: A Cross-Sectional Multicentric Study

**DOI:** 10.1155/2023/3002034

**Published:** 2023-05-15

**Authors:** Radwa R. Hussein, Enji Ahmed, Asmaa Abou-Bakr, Ayman A. El-Gawish, Abou-Bakr E. Ras, Dalia M. Ghalwash

**Affiliations:** ^1^Oral Medicine and Periodontology, Faculty of Dentistry, Ain Shams University, Cairo, Egypt; ^2^Oral Medicine and Periodontology, Faculty of Dentistry, Cairo University, Giza, Egypt; ^3^Oral Medicine and Periodontology, Faculty of Dentistry, The British University in Egypt, El Sherouk City, Egypt; ^4^Otolaryngology Department, Faculty of Medicine, Benha University, Qalyoubya, Egypt

## Abstract

**Background:**

Coronavirus disease 2019 (COVID-19) has rapidly spread over the globe, and several oral symptoms have been documented. However, it is unclear whether these lesions are the result of coronavirus infection or are secondary symptoms of the patient's systemic illness. The aim of this study was to collect data from various hospitals on COVID-19 patients with oral involvement in order to highlight different oral changes that may be manifested in those patients.

**Methods:**

This observational cross-sectional multicenter study used an online questionnaire covering oral signs and symptoms that were believed to be related to COVID-19 patients who were hospitalized in different hospitals in Egypt.

**Results:**

94.3% of the 210 patients who participated in the current study developed oral symptoms. Altered taste sensation (56.2%), burning sensation (43.3%), and oral candidiasis (40%) were the most prevalent oral symptoms (34.4%) that were found in the studied sample.

**Conclusions:**

COVID-19 has a major influence on the oral cavity, with numerous oral symptoms that may impair quality of life. Thus, considering the need for support, pain control, and management for a better prognosis, the clinical dental evaluation of hospitalized patients with infectious diseases like COVID-19 should be addressed.

## 1. Introduction

The novel coronavirus disease 2019 (COVID-19) is a human-to-human transmitted disease caused by one of the coronaviruses, a vast family of viruses that may cause serious illnesses like SARS and MERS [1]. COVID-19 has been declared a pandemic by the World Health Organization (WHO), with most countries reporting large numbers of infected persons and deaths as of December 2019 [2].

COVID-19 can be transmitted in two ways: directly or indirectly. Coughing, sneezing, and droplet inhalation, as well as direct contact with oral, nasal, and ocular mucous membranes, can spread it indirectly through saliva; while coughing, sneezing, and droplet inhalation, as well as direct contact with oral, nasal, and ocular mucous membranes, can spread it directly [3].

Clinical signs and symptoms range from a complete lack of symptoms to mild flu-like symptoms to severe respiratory infection. Individuals with comorbidities such as diabetes, hypertension, and ischemic heart disease experience more severe symptoms [4]. However, a significant majority of COVID-19 individuals are asymptomatic or have modest symptoms, necessitating the use of a predictive measure in addition to or instead of these manifestations [5].

The oral cavity, which is vulnerable to SARS-CoV-2, is thought to be a potential location of human-to-human viral transmission as well as a source of COVID-19 symptoms [6]. Furthermore, because SARS-CoV-2 possesses mucotropic activity and the ability to dysregulate the immune system and create a cytokine storm, COVID-19 may cause oral mucosal ulcers and inflammation [7].

SARS-CoV-2 enters target cells by connecting to the cellular angiotensin-converting enzyme 2 (ACE2) receptor and then priming viral spike proteins with transmembrane protease serine 2. (TMPRSS2). ACE2 and TMPRSS2 are found in epithelial cells of the human tongue and gingiva, with a higher concentration in the dorsal tongue and fungiform papillae taste cells [8]. Salivary glands express ACE2 and TMPRSS2 in the submandibular, parotid, and minor salivary glands, implying that salivary glands could be a reservoir for asymptomatic infection and release viral particles via salivary ducts [9].

SARS-CoV-2 has been found in the saliva of COVID-19 patients with high viral loads on a regular basis. SARS-CoV-2 infection is thought to alter gustatory function and saliva secretion due to the particular expression of ACE2 and TMPRSS2 in taste cells and salivary glands [10].

SARS-CoV-2 can cause TNF-a-converting enzyme (TACE)-dependent shedding of the ectodomain of ACE2, which is linked to TNF-a production [11]. TNF-a is an inflammatory cytokine produced by macrophages and monocytes during acute inflammation. It is responsible for a variety of signaling events within cells, including cell necrosis and apoptosis [12]. These findings imply that cellular signals induced by SARS-CoV-2's interaction with ACE2 are involved in the viral entry and also cause tissue damage [13].

Epithelial injury causes similar pathogenic features in the oral tissues in SARS-CoV-2, including ulcers, erosions, bullae, vesicles, pustules, fissured or depapillated tongue, macule, papule, plaque, pigmentation, halitosis, whitish areas, hemorrhagic crust, necrosis, petechiae, swelling, erythema, and Kawasaki-like features [14].

Lack of oral hygiene, opportunistic infections, stress, immunosuppression, vasculitis, and a hyperinflammatory response in COVID-19 patients were revealed to be predisposing factors for the emergence of oral lesions [15].

This virus is transmitted by air and, therefore, clinical practices with the production of contaminant aerosols are highly at risk. The use of minimally invasive therapies and bio-inspired systems such as laser and ozone and the administration of probiotics in nonsurgical dental treatment may be useful in reducing the risk of bacteremia and aerosol generation. This leads us to improve clinical, microbiological, and immunological parameters of fundamental importance in the context of global pandemic, where the reduction of bacterial load in aerosols becomes a pivotal point of the clinical practice [16].

## 2. Materials and Methods

Sample size calculation was not possible since the prevalence of COVID-19 was not yet estimated worldwide or in Egypt, and there was an incidence of new cases every day; this study was done at the peak time of the pandemic of COVID-19 in Egypt, so recruitment of patients was done via convenience sampling which is a type of nonprobability sampling that refers to the inclusion of the current available sample of COVID-19 patients suffering from any oral changes in the available time period.

This observational cross-sectional multicenter study was done on COVID-19 patients who were hospitalized in different hospitals in Egypt from January 2022 to April 2022.

The procedures were fully explained to the patients, and they signed informed consent. Individual patient's personal data and results were kept confidential by the filing system with passwords to protect them from being breached.

As clinical examination of the COVID-19 patients was not possible, and to avoid selection biases caused by convenience sampling, all participants had volunteered to reduce the risk of bias. People diagnosed with positive COVID-19 infection, verified by reverse transcriptase PCR (RT-PCR), and isolated at several Egyptian hospitals were included in the study.

We utilized Google Forms to develop an online questionnaire with different symptoms that, based on the evidence, could be linked to COVID-19. Patients' information was collected via an electronic survey, which was completed by internal residents in various Egyptian hospitals.

Patients had been informed about the different oral changes that appeared in some cases in association with the emerging COVID-19 pandemic to raise the awareness towards their oral hygiene.

## 3. Patient's Selection

Inclusion criteria were adults of age 20 years and above, patients with any underlying systemic diseases and medically free patients, patients with laboratory-confirmed COVID-19 infection via PCR (polymerase chain reaction), hospitalized patients, and patients who received an oxygen therapy.

Exclusion criteria were nonhospitalized patients, patients in the intensive care units, patients who had not received oxygen therapy, and patients who refused to participate in the study.

### 3.1. Questionnaire Tool

The questionnaire consisted of 3 sections and a total of 8 questions. The 1st section (demographic data) was regarding the age and sex. The 2nd section (medical condition) included all the chronic diseases that the patient might have, drugs, if the patients were under oxygen therapy, and its type. The 3rd section (oral manifestation) included all the oral lesions or changes that the patient could suffer from and also the site of these lesions.

## 4. Ethical Approval and Consent to Participate

This multicentric cross-sectional study was approved by the Research Ethics Committee, Faculty of Dentistry, the British University in Egypt, and written informed consent was obtained from all patients.

## 5. Results

Table 1 represents descriptive data for 210 patients diagnosed with positive COVID-19 infection having different oral manifestations including medical conditions and drugs taken.

We had 27 (12.9%) patients with age range from 20 to 29 years, 25 (11.9%) patients with age range from 30 to 39 years, 37 (17.6%) patients with age range from 40 to 49 years, 43 (20.5%) patients with age range from 50 to 59 years, and 78 (37.1%) patients who were above 60 years. 119 (56.7%) patients were male, while 91 (43.3%) were female.

Regarding medical conditions, 62 (29.5%) patients were medically free, while 70.5% had different medical conditions including diabetes mellitus, hypertension, chronic heart disease, liver disease, chronic kidney disease, immunologic disorders, blood disorders, thyroid diseases, allergic conditions (including bronchial asthma), and malignancy. The highest significant medical condition was diabetes mellitus in 102 patients with a prevalence of 48.6%.

100% of patients were under oxygen therapy, 150 (71.4%) were using C-PAP, and 60 (28.6%) were using oxygen masks.

100% of patients were taking medications including corticosteroids, systemic antibiotics, systemic antifungals, immunosuppressive drugs, and other miscellaneous drugs.

**Figure 1:**
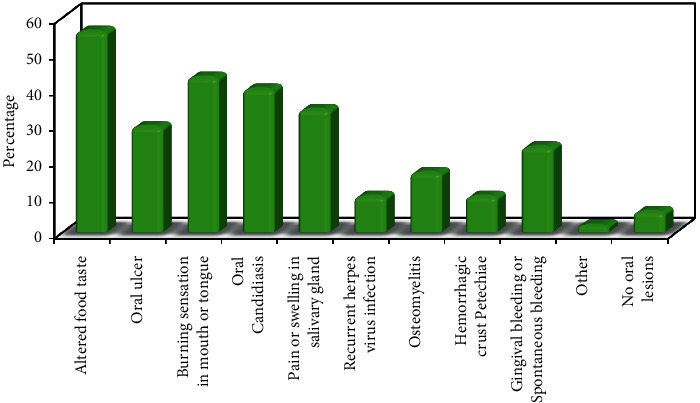
Distribution of the studied cases according to the patient suffering from any of the mentioned oral manifestations (*n* = 210).

Only 94.3% of patients had oral manifestations. The most prevalent oral manifestations were altered food taste in 118 (56.2%) patients, burning sensation in 91 (43.3%) patients, oral candidiasis in 84 (40%) patients, pain or swelling in the salivary glands in 72 (34.3%) patients, oral ulcers in 62 (29.5%) patients, and gingival bleeding or spontaneous bleeding in 50 (23.8%) patients, while the least prevalent oral manifestations were osteomyelitis in 35 (16.7%) patients, recurrent herpes virus infection in 21 (10%) patients, hemorrhagic crust and petechiae in 21 (10%) patients, or others in 4 (1.9%) patients, as shown in Figure 1.

**Figure 2:**
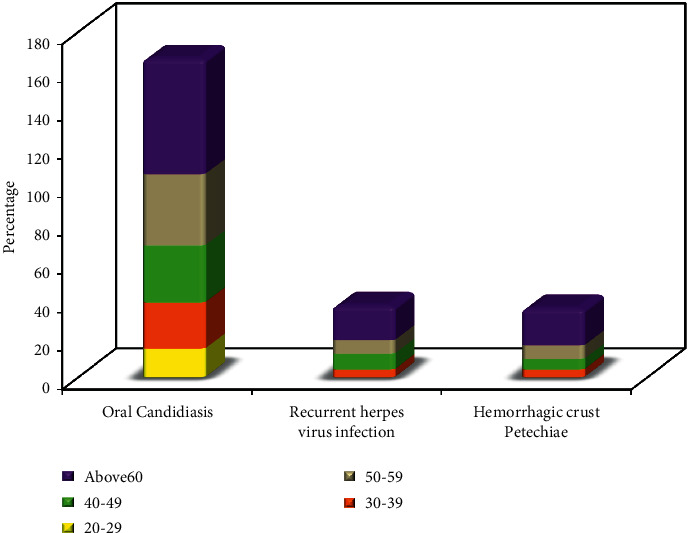
Relation between age and oral manifestation (*n* = 210).

The relation between age and oral manifestation (*n* = 210) as in Figure 2 shows that oral candidiasis, recurrent herpes virus infection, and hemorrhagic crust and petechiae were significantly high among the oldest age group (above 60 years) with a percentage of 60.3%, 17.9%, and 19.2%, respectively.

**Figure 3:**
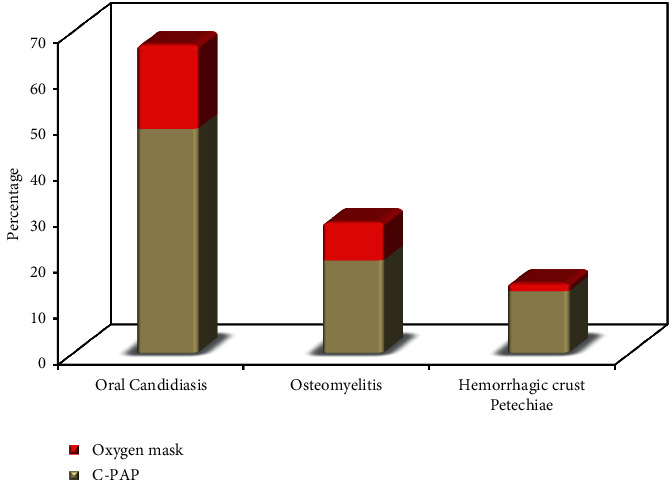
Relation between type of oxygen therapy and oral manifestation (*n* = 210).

Oral candidiasis was more prevalent in patients on C-PAP oxygen therapy (73 (48.7%)) than in those who were on oxygen masks (11 (18.3%)) with a high statistical significance with *p* value <0.001. Osteomyelitis was more prevalent in patients on C-PAP oxygen therapy (30 (20%)) than in those who were on oxygen masks (5 (8.3%)) with a high statistical significance with *p* value 0.040. Hemorrhagic crust and petechiae were more prevalent in patients on C-PAP oxygen therapy (20 (13.3%)) than in those who were on oxygen masks (1 (1.7%)) with a high statistical significance with *p* value 0.011, as shown in Figure 3.

## 6. Discussion

Currently, a safe pharmacological drug to combat COVID-19 has not been created yet, and the possible ones are associated with a variety of side effects, including oral lesions [17].

Furthermore, COVID-19 infection with the related therapeutic measures may contribute to negative oral health outcomes, such as opportunistic fungal infections, recurrent herpes simplex viral infection, fixed drug eruptions, unspecific oral ulcers, xerostomia, dysgeusia, and gingivitis resulting from an impaired immune function in a susceptible oral mucosa [18].

The emergence of these oral lesions could be explained by a variety of theories. First, the virus may interact with oral mucosal cells directly or indirectly [19]. According to a recent study, interactions between the virus and host epithelial cells may impair tissue integrity and cause the lesion [7]. Second, unfavorable reactions to medications used to treat viral infections might cause lesions. Herpes simplex virus and candida infection, xerostomia, nonspecific ulceration, and gingivitis are all possible side effects of several medications [15]. Third, lesions could also be caused by immune dysregulation and coinfection by opportunistic fungi, bacteria, or other types of viruses mainly related during the hospitalization period. Finally, it has been hypothesized that the oral lesions could be related to psychological factors such as work-related stress or limited social interaction [20].

Several studies have found that long durations of hospitalization increase the likelihood of acquiring oral lesions, emphasizing the significance of multidisciplinary care during this time, as well as supportive treatment of patients at home [15]. Despite the fact that oral lesions appear and develop at the same time as the signs and symptoms of COVID-19, the medical and dentistry communities are divided on the relevance of these lesions in the diagnosis of COVID-19 [21]. Mouth tissues have been reported to be the first to become infected with SARS-CoV-2, and oral lesions could theoretically be the first indications of COVID-19. If this hypothesis is validated, dentists will play a crucial role in the early detection of the disease, and they will be able to refer suspected SARS-CoV-2 patients for testing and treatment [19].

In the present study, male patients (56.7%) had a higher prevalence of oral manifestations than females (43.3%), which was consistent with prior research [20–22].

Oral symptoms were more common among our subjects over the age of 60, which was consistent with prior investigations [20, 22].

In the present study, most patients with different oral manifestations (94.3%) suffered from various medical conditions including diabetes mellitus, hypertension, chronic heart diseases, liver disease, chronic kidney disease, immunologic disorders, blood disorders, thyroid diseases, allergic conditions (including bronchial asthma), and malignancy and this was in accordance with several studies that reported similar underlying medical conditions [22, 23].

Altered taste sensation was found in 118 COVID-19 patients (56.2%), which is in line with previous studies [23, 24].

SARS-CoV-2 primarily exploits angiotensin-converting enzyme 2 (ACE2) receptors to gain access to cells, particularly those in the lower respiratory system. SARS-CoV-2 may infect nasal and oral mucosal cells on its way to that destination [25], which could explain the emergence of taste and smell dysfunctions early in the disease [26].

Although the cause of taste disorders in COVID-19 individuals is unknown, numerous theories have been proposed. Finsterer and Stollberger [27] speculated that rhinitis triggers could cause a local inflammatory reaction, which could impair taste buds' normal function. In patients with COVID-19, however, the presence of signs and symptoms related to nasal mucosal inflammation is not required for taste impairment. Taste abnormalities may occur before these signs, and numerous studies have found that taste disorders are more common than rhinorrhea, or that there is no link between the two [27, 28].

Burning sensation was present in 43.3% in our study which was in accordance with previous studies [29, 30]. Several factors can cause a burning feeling in the mouth (for example, candidal infection, dry mouth, oral ulcers, or drug-induced) [30].

Among the etiological factors linked to burning mouth syndrome (BMS), a number of psychological issues have received specific attention [28]. As a result of their anxiety, depression, low stress tolerance, and high degrees of neuroticism, these individuals are hypersensitive to any event that could bring them more stress [28, 31]. The finding that BMS is frequently linked to some life experience that emotionally destabilises the patient, thereby precipitating the onset of symptoms, is one example of this [31].

Oral candidiasis was present in 40% in the current study which was in line with earlier studies [22, 32]. Evidence suggests that viral infection might weaken the immune system, allowing secondary infections such as oral candidiasis to develop [30].

Many fatal COVID-19 cases were found to have bacterial and fungal coinfections, according to previous studies [25, 26].

Pain or swelling in the salivary gland was present in 34.4% in our study, and it was in agreement with previous studies [30, 33]. Effect of COVID-19 on the salivary glands manifested itself in two ways: first, patients experienced swelling or pain in the submandibular or parotid areas, and second, they experienced a dry mouth. These findings point to saliva's protective involvement in the oral cavity's ongoing cleaning and antiviral response to COVID-19 [34].

Respiratory infection incidence will then increase by enhancing virus adhesion and colonization and destroying the oral mucosa surfaces and airways, thus decreasing antimicrobial peptides and proteins [35]. In addition, the salivary glands can operate as a reservoir for a latent infection that might be reactivated later, resulting in chronic sialadenitis [36]; as a result, physicians should be more aware of any changes in saliva or salivary glands [32].

Oral ulcers were detected in 29.5% of COVID-19 patients in the present study, which was in accordance with the previous study [30, 37]. On both keratinized and nonkeratinized mucosae, aphthous-like lesions showed as many shallow ulcers with erythematous margins and yellowish-white pseudomembrane [14]. Increased levels of the tumour necrosis factor (TNF) can cause neutrophil chemotaxis to the oral mucosa and the formation of aphthous-like lesions in COVID-19 patients. Stress and immunosuppression caused by COVID-19 infection could also be factors in the development of such lesions in COVID-19 patients [15].

On the tongue, hard palate, and labial mucosa, ulcerative or erosive lesions developed as painful lesions with uneven borders. Several reasons have been proposed as explanations for the formation of ulcerative and erosive lesions, including drug eruption, vasculitis, or thrombotic vasculopathy owing to COVID-19 [4].

Gingival bleeding was present in 23.8% in the current study, and it was reported in previous studies [22, 29, 30]. COVID-19 is no exception to the rule of disregarding good dental hygiene when suffering from a debilitating illness. This is supported by a study that looked into COVID-19 problems in patients with poor dental health [38].

Osteomyelitis was detected among 16.7% of patients in the current study, which is a bone inflammatory condition that usually starts in the medullary cavity, quickly spreads to the Haversian systems, and extends to the periosteum of the affected area [39]. The pus that forms as a result of the infection affects the blood flow beneath the periosteum, causing ischemia and necrosis. Because of the wide blood supply, the existence of thin cortical plates, and the scarcity of medullary tissues, maxillary osteomyelitis occurs less frequently. The presence of fungus in the bone marrow promotes fungal growth by damaging the endothelial lining of vessels, resulting in vascular insufficiency, which finally leads to bone necrosis and fungal osteomyelitis. The diagnosis of fungal osteomyelitis is quite difficult [40]. However, people with COVID-19 have been documented to develop osteomyelitis of additional bones (foot, palm, and umbilical cord) [41].

Maxillofacial osteomyelitis in COVID-19 individuals has a long-term course, with chronic progressive and atrophic processes predominating. The efficiency of comprehensive treatment was found to be below in virtually all cases related to metabolic problems in the maxillary bone. The ineffectiveness of treatment in many circumstances, as seen in the aforementioned patient, necessitates the intensification of rehabilitation measures aimed at improving the general state of patients, followed by the rebuilding of the faults. However, we must not overlook the negative effects of pharmaceuticals used to treat patients in the early and late phases of the disease (corticosteroids, immunosuppressants, and interleukin-6 receptor inhibitors) [42].

Petechiae were found in 10% of cases on the lower lip, palate, and oropharynx mucosa in the current study. Petechiae have previously been documented on the lower lip, palate, and oropharynx mucosa. Petechiae have been linked to thrombocytopenia caused by COVID-19 infection or the prescribed medicine [24, 43].

Recurrent HSV was reported in 10% in our study which was reported in previous studies [24, 43]. It is claimed that HHV plays a role in the worsening of oral diseases and that it has a synergistic effect with bacterial etiological factors. Members of the HHV family can be found in a variety of oral diseases, including pulpitis, periapical periodontitis, periodontitis, and peri-implantitis [44]. HHV-encoded microRNAs are frequently found in inflammatory pulpal and periodontal tissues, implying viral reactivation. Viral microRNAs, by suppressing various host transcripts, can aid in the evasion of host defensive responses, such as antiviral responses and the clearance of virus-infected cells, by interfering with antigen presentation [44].

In the present study, the most common site of appearance of oral lesions was buccal mucosa (45.2%), and this was in agreement with previous reports [15, 43, 45], regarding the palate [45], followed by the lip mucosa (38.6%) [43] and buccal mucosa (32.9%) [22, 23].

Probiotic therapy [46] in management of oral soft and hard tissues has recently gained attention in the scientific community due to lack of side effects often associated with antibiotic use [47]. Despite its effectiveness, probiotics have prompted various concerns in recent years. The safety of live microorganisms, in particular, should be considered, especially when they are given to vulnerable patients like the elderly and immunocompromised people [48, 49].

New products based on nonviable probiotics, such as paraprobiotics (tyndallized probiotics) and postbiotics, have been proposed in response to these limitations. Paraprobiotics, in particular, are inactivated microbial cells that provide a benefit to the consumer while posing no health risk; they can regulate both adaptive and innate immune systems, exert an antagonistic effect against pathogens, and have anti-inflammatory, antiproliferative, and antioxidant properties, with maintenance of eubiosis and avoiding hard and soft tissue injuries [48]. Postbiotics, which include any chemical released by or created through the metabolic activity of the microorganism without including the living bacteria themselves [50], should not be confused with probiotics and paraprobiotics [51].

## 7. Conclusions

The present study reveals that COVID‐19 infection has a significant impact on the oral health. Altered taste sensation followed by burning sensation, oral candidiasis, salivary gland‐related symptoms, and oral ulcers occurred with high prevalence in COVID‐19 patients. The clinical oral examination of patients with COVID-19 should be prioritized in order to detect any oral complications as early as possible to accomplish proper and timely management, which accordingly improves the patient's quality of life.

Further clinical studies with detailed patient's history and larger sample sizes are required to validate our results and clarify the full impact of COVID‐19 on oral health.

## Figures and Tables

**Table 1:** Different descriptive data for all 210 patients diagnosed with positive COVID-19 infection.

Descriptive data	No. (%)
Medical conditions	
Diabetes	102 (48.6%)
Hypertension	83 (39.5%)
Heart disease	33 (15.7%)
Liver disease	3 (1.4%)
Kidney disease	18 (8.6%)
Immunologic disorder	6 (2.9%)
Blood disorder	2 (1.0%)
Thyroid problem	4 (1.9%)
Malignancy	4 (1.9%)
Asthma	6 (2.9%)
Allergic diseases	3 (1.4%)
Medically free	62 (29.5%)

Drugs used	
Corticosteroid therapy	176 (83.8%)
Systemic antibiotic	188 (89.5%)
Systemic antifungal	15 (7.1%)
Immunosuppression drugs	20 (9.5%)
Others	54 (25.7%)

Is the patient under oxygen therapy?	
Yes	210 (100%)
No	0 (0%)

If yes, which type of oxygen therapy	
C-PAP	150 (71.4%)
Oxygen mask	60 (28.6%)

## Data Availability

The data that support the findings of this study are available from different hospitals in Egypt, and restrictions apply to the availability of these data, which were used under license for the current study and so are not publicly available. Data, however, are available from the corresponding author upon reasonable request.
